# Factors associated with hyperresponsiveness to adenosine 5’‐monophosphate in healthy subjects

**DOI:** 10.1111/all.13864

**Published:** 2019-06-07

**Authors:** Claire Alette Cox, Judith M. Vonk, Huib A.M. Kerstjens, Maarten van den Berge

**Affiliations:** ^1^ Department of Pulmonary Diseases University Medical Center Groningen University of Groningen Groningen The Netherlands; ^2^ University Medical Center Groningen Groningen Research Institute for Asthma and COPD University of Groningen Groningen The Netherlands; ^3^ Department of Epidemiology University Medical Center Groningen University of Groningen Groningen The Netherlands

To the editor

Airway hyperresponsiveness (AHR) is used to diagnose asthma. AHR can be assessed with a direct (eg, methacholine) or an indirect (eg, adenosine 5’‐monophosphate (AMP)) test. Direct tests induce airway constriction by stimulating airway smooth muscle contraction. Indirect tests provoke mast cells and other inflammatory cells to release mediators, that cascade into airway constriction. AHR can be defined as the concentration of the stimulus that causes a 20% drop in forced expiratory volume in the first second (FEV_1_)(PC_20_). Although healthy subjects are expected to be unresponsive to AMP,^1^ in a group of 108 healthy controls we observed 8 (7.4%) subjects with AHR to AMP (PC_20_AMP ≤160 mg/mL). We therefore investigated which factors associate with AHR to AMP in these healthy subjects.

The NORM database (approved by the ethical committee of our hospital, NCT00848406, see Data S1) consists of healthy never or current smokers (>10 cigarettes per day). Subjects had no respiratory complaints (ie, cough or dyspnoea) nor any doctor's respiratory diagnosis, in the past or present. They presented with normal lung function (FEV_1_ >80%_predicted_, FEV_1_/FVC >70%, and <10% increase in FEV_1_%_predicted_ after 400 μg salbutamol) and the absence of AHR to methacholine (PC_20_methacholine >19.8 mg/mL; Table 1). To investigate which factors associate with AHR to AMP, we compared subjects with AHR to AMP to those without (≤160 mg/mL vs >160 mg/mL, Table 1) with a chi‐square or Mann‐Whitney U test. From each group of variables, we entered in a multivariate logistic regression model the variable which increased the model's adjusted‐*R* ^2^ most (univariate *P* < 0.01 and mutual Spearman's correlation <|0.7|; Table 2, SPSS version 22).

**Table 1:** Univariate analysis comparing subject with and without airway hyperresponsiveness to AMP

	AMP > 160 mg/mL (n = 100)	AMP ≤ 160 mg/mL (n = 8)	*P*‐value
Gender (male‐female)	57‐43	4‐4	7.0 × 10^−1^
Age (years)	40.50 (22.0; 55.0)	43.00 (25.5; 57.5)	5.4 × 10^−1^
Positive skin prick test (no‐yes)	66‐34	6‐2	5.9 × 10^−1^
Allergic rhinitis (self‐reported) (no‐yes)	86‐14	7‐1	9.1 × 10^−1^
Smoking status (never‐current)	55‐45	0‐8	2.8 × 10^−3^ *
Packyears (years)	0.3 (0.0; 10.5)	22.55 (7.0; 46.8)	6.0 × 10^−4^ *
Smokers ‐ Packyears (years)	13.3 (2.9; 25.0)	22.55 (7.0; 46.8)	1.6 × 10^−1^
Smokers ‐ Equivalent number of cigarettes/day	14.0 (10.0; 18.0)	19.5 (17.3; 23.8)	9.5 × 10^−3^ *
Blood eosinophils (%)	2.4 (1.4; 3.4)	2.6 (2.0; 5.1)	2.7 × 10^−1^
Preprovocation sputum eosinophils (categorized) (absent (<1%)—present (≥1%)—no sample)	60‐7‐33	3‐4‐1	1.0 × 10^−3^ *
Preprovocation sputum eosinophils (%)^a^	0.0 (0.0; 0.4)	1.0 (0.7; 2.9)	2.7 × 10^−3^ *
FEV_1%_predicted	109 (101; 114)	100 (90; 110)	7.2 × 10^−2^
FEF_25%_predicted	102 (92; 118)	104 (94; 119)	6.4 × 10^−1^
FEV_1_/FVC (%)	79.33 (76.1; 84.1)	74.98 (71.7; 83.6)	1.5 × 10^−1^
R_5_ (kPa/sL)	0.30 (0.2; 0.4)	0.36 (0.3; 0.5)	9.1 × 10^−2^
R_20_ (kPa/sL)	0.28 (0.2; 0.3)	0.28 (0.2; 0.3)	8.8 × 10^−1^
FEF_50%_predicted	89 (77; 105)	72 (67; 83)	2.3 × 10^−2^
FEF_75%_predicted	73 (59; 94)	52 (42; 64)	7.7 × 10^−3^ *
FEF_25‐75%_predicted	88 (74; 103)	68 (63; 79)	1.1 × 10^−2^
R_5_‐R_20_ (kPa/sL)	0.02 (0.0; 0.0)	0.11 (0.0; 0.1)	2.7 × 10^−3^ *
AX (kPa/L)	0.15 (0.1; 0.2)	0.35 (0.2; 1.1)	2.8 × 10^−3^ *
F_res_ (L/s^-1^)	9.77 (8.39; 11.42)	14.91 (10.71; 19.65)	4.8 × 10^−3^ *
TL_CO_ %predicted	92 (83; 101)	81 (75; 86)	7.4 × 10^−3^ *
K_CO_ %predicted	97 (89; 107)	92 (88; 94)	9.9 × 10^−2^
COPD Control Questionnaire (CCQ)	0.20 (0.1; 0.4)	0.60 (0.4;1.2)	4.7 × 10^−3^ *
Asthma Control Questionnaire (ACQ)	0.00 (0.0; 0.0)	0.00 (0.0;0.1)	2.7 × 10^−2^
St. George Respiratory Questionnaire (SGRQ)	1.93 (1.1; 4.4)	7.65 (2.3; 16.7)	1.0 × 10^−2^ *

Note

Data are presented as count or median (inter quartile range (IQR)). Binominal data were analyzed with a chi‐square test, while continuous data were analyzed with a Mann‐Whitney *U* test.

Abbreviations: AMP= adenosine 5’‐monophosphate; FEF_25_, forced expiratory flow at 25% of FVC; FEF_50_, forced expiratory flow at 50% of FVC; FEF_75_, forced expiratory flow at 75% of FVC; FEF_25‐75_, forced expiratory flow at 25% to 75% of FVC; R_5_, resistance to 5 Hz; R_20_, resistance to 20 Hz; R_5_‐R_20_, difference in resistance to 5 Hz and 20 Hz; AX, reactance area; F_res_, resonance frequency; TL_CO_, transfer factor for carbon monoxide; K_co_, TL_CO_ corrected for the alveolar volume; FEV_1_, forced expiratory volume in the first second; FVC, forced vital capacity.

a n = 74, 67 without AHR and 7 with AHR.

*P ≤ 0.01.

**Table 2:** Multivariate logistic regression model predicting airway hyperresponsiveness to AMP

	B	Standard error	*P*‐value
Equivalent number of cigarettes/day^a^	0.224	0.134	9.6 × 10^−2^
R_5_‐R_20_ b	0.299	0.147	4.1 × 10^−2^
TL_CO_ %predicted	−0.060	0.059	3.1 × 10^−1^
Preprovocation sputum eosinophils			
Absent(<1%)	‐	‐	‐
Present (≥1%)	3.016	1.655	6.8 × 10^−2^
Missing (no sample)	−4.084	3.074	1.8 × 10^−1^
St. George Respiratory Questionnaire (SGRQ)	0.544	0.256	3.4 × 10^−2^

Note

The logistic regression model was obtained by including the variable which improved the model's adjusted‐*R* ^2^ most (univariate *P *≤ 0.01 and mutual Spearman's correlations <|0.7|), from each group of variables in Table 1. This model has a Cox & Snellen *R* ^2^ of 30.4% and 96.3% correctly predicted (SPSS version 22).

Abbreviations: R_5_‐R_20_, difference in resistance to 5 and 20 Hz; TL_CO_, transfer factor for carbon monoxide.

a Based on all subject (never and current smokers); current and never smokers.

b incorporated in the multivariate regression model on 0.01 scale.

We expected a relation with allergies based on a skin prick test (*P* = 0.59) and self‐reported allergic rhinitis (*P* = 0.91), but this relation was absent. Two samples had sputum eosinophils above the commonly used threshold of 3%. Therefore, we analyzed the percentage of sputum eosinophils categorized as <1%, ≥1%, and missing (only 74 subjects were able to expectorate sputum). AHR was significantly associated with sputum eosinophils (Table 1). The multivariate model (Table 2) shows that AHR is more likely in subjects with ≥1% sputum eosinophils compared to subjects with <1% sputum eosinophils, but equally likely in subjects with a missing sputum sample compared to those with <1% sputum eosinophils. This indicates that ≥1% sputum eosinophils in a healthy subject associates with AHR to AMP. This aligns with earlier observations in asthma and allergic rhinitis that sputum eosinophils predict AHR to AMP.^2^ We now show the association in healthy subjects.

Strikingly, all subjects with AHR to AMP were current smokers. Smoking status was significantly different (*P *< 0.01) between subjects with and without AHR and smokers with AHR smoked more cigarettes per day than smokers without AHR (*P* < 0.01). Previously reported healthy subjects with AHR to AMP were also all current smokers.^3^ Furthermore, smoking is associated with AMP sensitivity in patients with chronic obstructive pulmonary disease (COPD),^3^ as COPD smokers had a significantly lower PC_20_ AMP compared to COPD nonsmokers. Based on these findings, we speculate that AHR to AMP in healthy subjects is affected by cigarette smoke‐induced presence of mast cells, the primary target for AMP. Cigarette smoke irritates epithelial cells, initiating the release of pro‐inflammatory cytokines.^4^ These trigger mast cell infiltration and proliferation, resulting in increased numbers of mast cells in the airway's submucosa.^5^ Cigarette smoke also causes the reduction of epithelial integrity which facilitates the cigarette smoke to reach the mast cells.^6^ As AMP provocation initiates bronchoconstriction through mediators released by mast cells,^7^ healthy smokers may have AHR to AMP.

Next to smoking, AHR to AMP strongly associated with small airways function. Univariately, AHR to AMP associated with lower forced expiratory flow at 50%, 75%, and 25%‐75% of the forced vital capacity (FVC) (FEF_50_, FEF_75_, and FEF_25‐75_, respectively), a higher resistance in the small airways (difference in resistance to 5 Hz and 20 Hz (R_5_‐R_20_)), and lower lung compliance (increased resonance frequency (F_res_) and reactance area (AX); all *P* < 0.03, Table 1). Multivariately, small airways function, in terms of R_5_‐R_20_, was independently associated with AHR (Table 2). Previously, small airways function has been suggested to explain why some patients present with respiratory complaints, despite a normal FEV_1_. It is possible that small airways anomalies are already important before respiratory complaints arise, as AHR to AMP might occur earlier than AHR to methacholine. Michils *et al* 8 proposed that a discrepancy in the response to methacholine and AMP arises from the different areas the provocative agents trigger. They showed that AHR to methacholine originates more centrally in the lungs, where the targets for methacholine, the muscarinic receptors, have a higher density. By contrast, AHR to AMP originates from the most peripheral lung zone (pre‐acinar airways), where in its submucosa mast cells are predominantly localized. Involvement of the most peripheral lung zones is furthermore indicated by the association between PC_20_AMP ≤160 mg/mL and a lower diffusion capacity (T_L,CO_), in both univariate and multivariate analysis (Table 1, 2). Diffusion capacity indirectly reflects the small airways via its association with acinar airways dysfunction (increased S_acin_).^9^

Finally, AHR to AMP associated with a lower quality of life. Univariately, hyperresponsive subjects scored higher on the St. George respiratory questionnaire (SGRQ) and asthma and COPD control questionnaires (ACQ and CCQ, respectively; Table 1). Nevertheless, all scores were below the clinically used thresholds of ≤25, ≤1.5, and ≤1, respectively, indicating that our subjects have few symptoms. Multivariately, the SGRQ was independently associated with AHR to AMP (Table 2) indicating an association with lower quality of life. From this, we speculate that AHR to AMP in healthy subjects may be an early predictor for development of pulmonary complaints.

In conclusion, in a group of healthy never and current smokers, eight subjects (7.4%) expressed AHR to AMP (PC_20_AMP ≤160 mg/mL). These subjects were current smokers with a higher cigarette consumption compared to subjects without AHR. Furthermore, AHR to AMP associated with a reduced small airways function, higher sputum eosinophil levels, and a lower quality of life. Further research should concentrate on whether AHR to AMP in healthy smokers indicates the onset of respiratory disease development.

## CONFLICT OF INTEREST

C.A. Cox, J.M. Vonk, and H.A.M. Kerstjens have nothing to disclose. Maarten van den Berge reports grants paid to the University from Astra Zeneca, TEVA, GSK, Chiesi, outside the submitted work.
