# Supplementary material for: Factors associated with hyperresponsiveness to adenosine 5’‐monophosphate in healthy subjects
Source: Allergy. 2019 Jun 7;74(11):2268–70. doi: 10.1111/all.13864 (PMC6899818; doi:10.1111/all.13864)
Supplement: Supplementary file 1 [file ALL-74-2268-s001.docx]

Factors associated with hyperresponsiveness to adenosine 5’‑monophosphate in healthy subjects

# Supplement

The NORM study (clinicaltrails.gov NCT00848406) was conducted to obtain a control group for asthma and COPD studies conducted in our department. Subjects were selected to be either current or never smoker, and either over or under 40 years of age in order to be able to select a representative control group. For adequate comparison of these subjects to those in the asthma or COPD studies , the NORM study meticulously characterized its participants with pulmonary function tests (spirometry pre- and post-salbutamol, impulse oscillometry, body plethysmography, CO-diffusion, and provocation with methacholine and AMP), FeNO, skin-prick allergy tests, blood tests, expectorated sputum, HRCT-scans, health questionnaires (ACQ, CCQ, BHQ, St. George respiratory questionnaire, Fagerstrom Questionnaire, Beck Depression Inventory overall score, and RAND36), biopsies and nasal brushes. Furthermore, ethnicity, educational level, personal- and family medical history, and medication use were reported. All subjects provided written informed consent. The NORM study was approved by the ethics committee of the University Medical Center Groningen.
